# Deoxygenative *gem*-difluoroolefination of carbonyl compounds with (chlorodifluoromethyl)trimethylsilane and triphenylphosphine

**DOI:** 10.3762/bjoc.10.32

**Published:** 2014-02-06

**Authors:** Fei Wang, Lingchun Li, Chuanfa Ni, Jinbo Hu

**Affiliations:** 1 Key Laboratory of Organofluorine Chemistry, Shanghai Institute of Organic Chemistry, Chinese Academy of Sciences, 345 Ling-Ling Road, Shanghai 200032, China

**Keywords:** (chlorodifluoromethyl)trimethylsilane, difluorocarbene, *gem*-difluoroolefin, organo-fluorine, Wittig reaction, ylide

## Abstract

**Background:** 1,1-Difluoroalkenes cannot only be used as valuable precursors for organic synthesis, but also act as bioisosteres for enzyme inhibitors. Among various methods for their preparation, the carbonyl olefination with difluoromethylene phosphonium ylide represents one of the most straightforward methods.

**Results:** The combination of (chlorodifluoromethyl)trimethylsilane (TMSCF_2_Cl) and triphenylphosphine (PPh_3_) can be used for the synthesis of *gem*-difluoroolefins from carbonyl compounds. Comparative experiments demonstrate that TMSCF_2_Cl is superior to (bromodifluoromethyl)trimethylsilane (TMSCF_2_Br) and (trifluoromethyl)trimethylsilane (TMSCF_3_) in this reaction.

**Conclusion:** Similar to many other Wittig-type *gem*-difluoroolefination reactions in the presence of PPh_3_, the reaction of TMSCF_2_Cl with aldehydes and activated ketones is effective.

## Introduction

The synthesis and application of selectively fluorinated organic molecules have attracted much interest from both organic chemists and biochemists because fluorine can endow these molecules with unique chemical, biological and physical properties [1–3]. 1,1-Difluoroalkenes have been frequently used in the design of potential enzyme inhibitors [4–6], since difluoromethylene functionality (CF_2_) is known to be isosteric and isopolar to an oxygen atom [7–9], and the *gem*-difluorovinyl functionality is believed to be a bioisostere for a carbonyl group [10]. More commonly, 1*,*1-difluoroalkenes, which are highly electrophilic towards many nucleophiles at the terminal difluoromethylene carbon [11], are used as valuable precursors of di- and trifluoromethyl compounds [10,12], monofluoroalkenes [13], monofluorinated heterocycles [14–15], carboxylic acids and esters [16]. Consequently, these relevant applications of 1,1-difluoroalkenes have led to many efforts to develop *gem*-difluoroolefination methods including β-elimination of functionalized difluoromethyl compounds, transition metal catalysed coupling reactions with *gem*-difluorovinylation reagents, and deoxygenative *gem*-difluoroolefination of carbonyl compounds [17–18]. Among these methods, the latter one has been studied with several named reactions, for example Wittig, Horner–Wadsworth–Emmons, and Julia–Kocienski reactions.

**Scheme 1:**
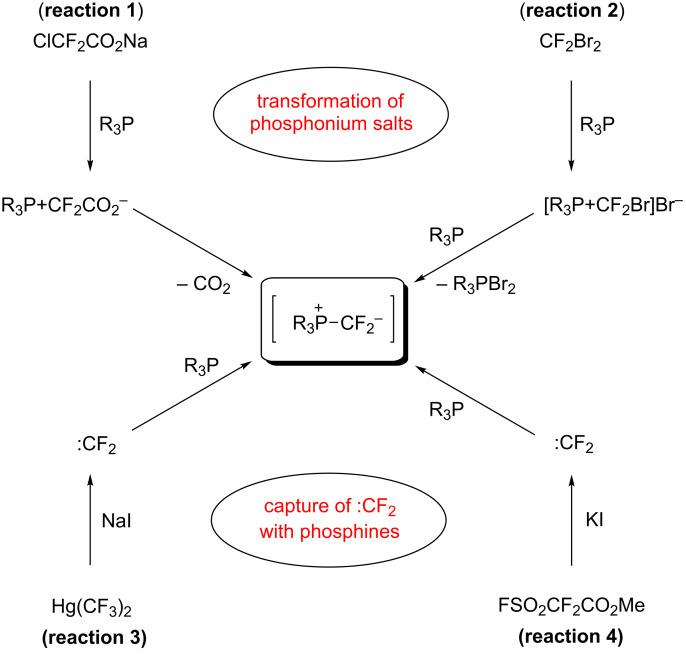
Various procedures for the generation of difluoromethylene phosphonium ylide [19–25].

In the Wittig *gem*-difluoroolefination, the reaction is believed to proceed via an undetected difluoromethylene phosphonium ylide, which can be generated in situ either by the transformation of a difluorinated phosphonium salt or by the reaction between difluorocarbene (:CF_2_) and a phosphine (Scheme 1) [19–26]. In 1964, Fuqua and co-workers first reported the difluoromethylenation of aldehydes by using ClCF_2_CO_2_Na/PPh_3_ [19]. In 1967, Burton and Herkes suggested that the ylide intermediate involved in the olefination process was more likely to be formed by the decarboxylation of a difluorinated phosphonium salt rather than the combination of :CF_2_ and a phosphine (Scheme 1, reaction 1) [20]. Their suggestion is based on the accelerating effect of PPh_3_ on the thermal decomposition of ClCF_2_CO_2_Na and the unsuccessful capture of :CF_2_ with an alkene or alcohol during the olefination reaction [20]. Very recently, the successful preparation of (triphenylphosphonio)difluoroacetate (Ph_3_P^+^CF_2_CO_2_^−^) and its application in carbonyl *gem*-difluoroolefination by Xiao and co-workers [21] finally confirmed the mechanism proposed by Burton and others [19–20]. Burton and co-workers also developed another difluorocarbene-free approach using a 1:2 mixture of CF_2_Br_2_ and PPh_3_ or P(NMe_2_)_3_ to prepare the ylide intermediate (Scheme 1, reaction 2) [22–23]. Although the difluorocarbene/phosphine procedure for Wittig olefination has been put forward by Fuqua et al. as early as 1964 [19], the formation of difluoromethylene phosphonium ylide in such a way is quite rare [24–26]. Established examples include using bis(trifluoromethyl)mercury (Hg(CF_3_)_2_) under the promotion of NaI (Scheme 1, reaction 3) [24] and using methyl 2,2-difluoro-2-(fluorosulfonyl)acetate (MDFA) under the promotion of KI (Scheme 1, reaction 4) [25].

**Scheme 2:**
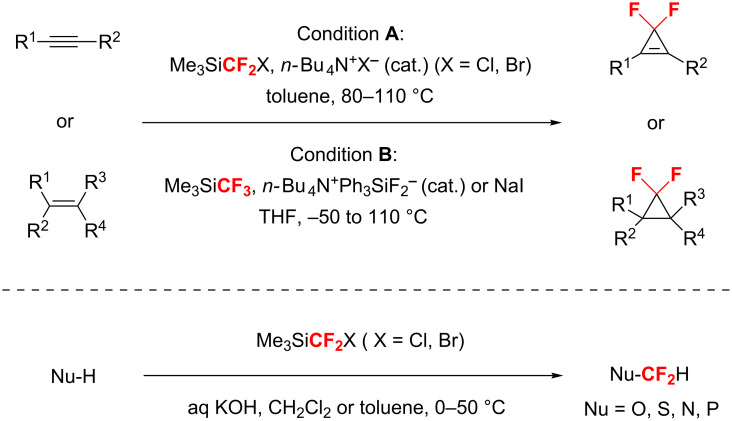
Difluoromethylenation of alkenes and alkynes and difluoromethylation of heteroatom nucleophiles with TMSCF_2_X [31–34].

Our group has focused on the development and application of new difluorocarbene reagents [27–34]. The Prakash group and we have identified that (halodifluoromethyl)trimethylsilanes (TMSCF_2_X, X = F, Cl, and Br) could serve as difluorocarbene sources under the activation of proper halide initiators or alkaline bases (Scheme 2) [31–34]. Recently, we have developed a relatively environmentally benign method to prepare TMSCF_2_Br, which can be used as a general carbene source for the difluoromethylenation of alkynes and alkenes and difluoromethylation of heteroatom nucleophiles [34]. In this paper, the novel preparation of TMSCF_2_Cl from TMSCF_2_Br and the application of the former in deoxygenative *gem*-difluoroolefination of carbonyl compounds via Wittig-type reaction are reported.

## Results and Discussion

**Scheme 3:**
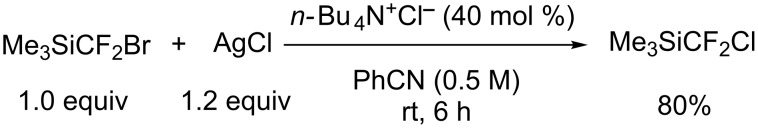
Bromo–chloro exchange reaction using AgCl.

(Halodifluoromethyl)trimethylsilanes including TMSCF_3_ (Ruppert–Prakash reagent), TMSCF_2_Cl, and TMSCF_2_Br are initially prepared by reductive silylation of ozone-depleting-substances bromotrifluoromethane (CF_3_Br) [35], bromochlorodifluoromethane (CF_2_BrCl) [36–37], and dibromodifluoromethane (CF_2_Br_2_) [36–37] with chlorotrimethylsilane (TMSCl). In recent years, Prakash and co-workers have discovered two Freon-free methods for the synthesis of TMSCF_3_ from fluoroform (CF_3_H), which paved the way for the synthetic applications of TMSCF_3_ [38–39]. Moreover, the preparation of TMSCF_2_Br either by fluoro–bromo exchange reaction of TMSCF_3_ [34] or by bromination of TMSCF_2_H [34,40] has also been disclosed. To obtain TMSCF_2_Cl, we tried the halogen exchange reaction of TMSCF_2_Br. When a 1:10 mixture of TMSCF_2_Br and TMSCl was heated in neat in the presence of 5 mol % of tetrabutylammonium chloride (TBAC) for 2 hours, ^19^F NMR spectroscopy analysis showed that the ratio of TMSCF_2_Cl to TMSCF_2_Br was 2.3:1, and prolonging reaction time could not improve the ratio. In view of the difficulty in separating TMSCF_2_Cl from the reaction mixture because of the approximate boiling points of TMSCF_2_Cl (~85 °C) [36–37] and TMSCF_2_Br (~105 °C) [36–37], other chloride sources were tried to achieve a full conversion of TMSCF_2_Br. Gratifyingly, when the reaction was performed in benzonitrile (bp ~190 °C) at 80 °C using a slight excess of silver chloride under the catalysis of TBAC, a full conversion of TMSCF_2_Br afforded TMSCF_2_Cl in 54% yield. Lowering the temperature to room temperature (rt) could improve the yield to 80% (Scheme 3). It is believed that the lower solubility of silver bromide than silver chloride in benzonitrile provides the driving force for this bromo–chloro exchange reaction.

At first, the olefination of 1-naphthaldehyde (**1a**) or benzaldehyde (**1b**) by using the combination of TMSCF_2_Cl and PPh_3_ was tried. Conceiving that the chloride ion might be necessary to promote the decomposition of TMSCF_2_Cl to release CF_2_ as reported, a catalytic amount of TBAC was used as the initiator. After heating a reaction mixture of aldehyde **1a**, TMSCF_2_Cl, PPh_3_, and TBAC in THF at 100 °C for 8 h, ^19^F NMR spectroscopy analysis showed that difluorinated alkene **2a** was formed in 69% yield (Table 1, entry 1). Surprisingly, it was found that in the absence of TBAC, PPh_3_ could be used both to promote the fragmentation of TMSCF_2_Cl and combine with the generated :CF_2_ (Table 1, entry 2). A rough comparison of the reaction temperatures showed that a lower temperature (rt) is detrimental to the olefination process, although the decomposition of TMSCF_2_Cl could occur to some extent (Table 1, entries 2 and 3).

**Table 1:** Condition screening of *gem*-difluoroolefination with TMSCF_2_X.

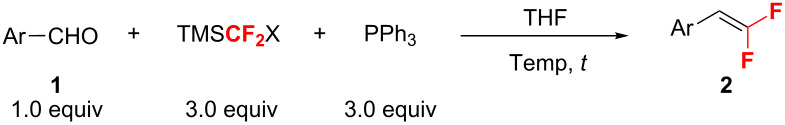							

Entry^a^	Ar	X	Initiator	Temp (°C)	*t* (h)	Conversion (%)^b^	Yield (%)^b^

1	1-naphthyl	Cl	TBAC (3 mol %)	100	8	100	69
2	1-naphthyl	Cl	none	70	10	100	59^c^
3	Ph	Cl	none	rt	4	35	0
4	Ph	Br	none	70	10	100	0
5	Ph	F	NaI (0.6 equiv)	70	10	<5	0
6	Ph	F	NaI (6.0 equiv)	110	10	<5	0

^a^Reactions were performed on 0.5 mmol scale in a pressure tube. ^b^Conversion of TMSCF_2_X and yields of **2** were determined by ^19^F NMR spectroscopy using PhCF_3_ as an internal standard. ^c^Isolated yield of **2a**.

**Scheme 4:**
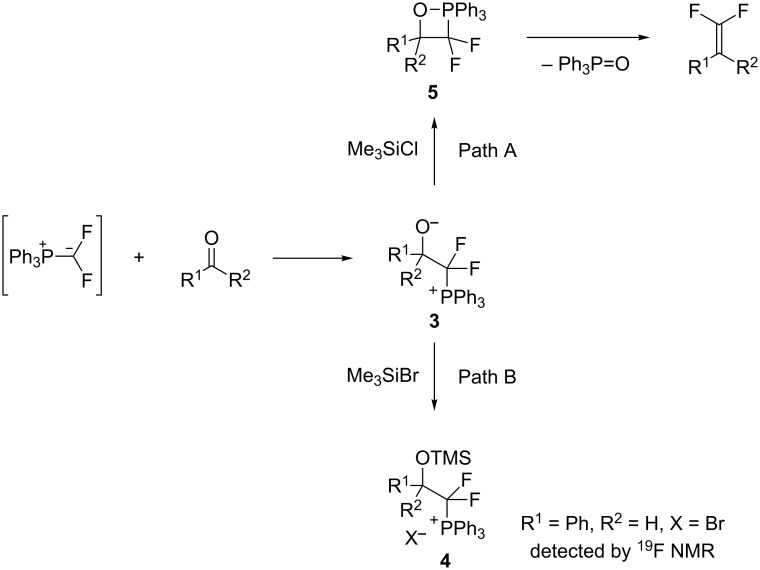
Proposed different reaction pathways of the difluorinated ylide in the presence of TMSCl and TMSBr.

Subsequently, the olefination of aldehyde **1b** with TMSCF_2_Br was examined. Unfortunately, the full consumption of TMSCF_2_Br did not afford any difluoroolefin **2b** (Table 1, entry 4). As determined by ^19^F NMR, besides the side product (difluoromethyl)triphenylphosponium bromide (δ −127.9, dd, ^3^*J*_P-F_ = 80 Hz, ^2^*J*_F-H_ = 47 Hz) as reported in the Wittig olefination with FSO_2_CF_2_CO_2_Me [25], a new product which was assigned as difluorinated phosphonium salt **4** (δ −88.8, ddd, ^2^*J*_F-F_ = 298 Hz, ^3^*J*_P-F_ = 97 Hz, ^3^*J*_F-H_ = 3.3 Hz, 1F; δ −106.6, ddd, ^2^*J*_F-F_ = 298 Hz, ^3^*J*_P-F_ = 101 Hz, ^3^*J*_F-H_ = 24 Hz, 1F) was detected as the major product (for details, see Supporting Information File 1). The formation of **4** is supposed to arise from a ready silylation of the addition intermediate betaine **3** by TMSBr. When TMSCF_2_Cl was used, TMSCl is not reactive enough to trap the betaine **3**, thus the oxaphosphetane **5** could be formed to give olefins and triphenylphosphine oxide (Scheme 4).

Finally, the olefination of aldehyde **1b** with TMSCF_3_ as the difluoromethylene source was tested. The results showed that no desired reaction took place when PPh_3_ and either substoichiometric or stoichiometric amounts of NaI were used (Table 1, entries 5 and 6). Although it has been known that TMSCF_3_ can be used in the difluoromethylenation of alkenes and alkynes initiated by NaI [33], we could not give a reasonable explanation for the failure of the current reaction.

**Figure 1:**
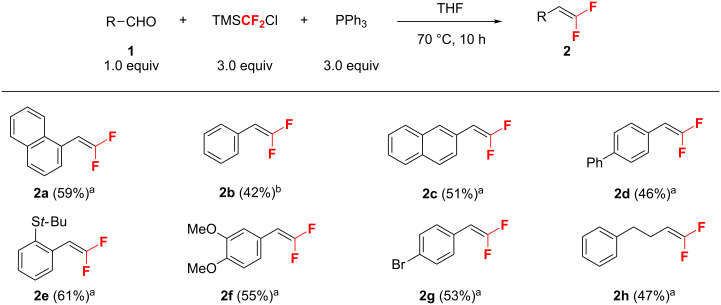
*gem*-Difluoroolefination of aldehydes. Reactions were performed on 0.5 mmol scale in a pressure tube. ^a^Isolated yield. ^b^Yield was determined by ^19^F NMR spectroscopy using PhCF_3_ as an internal standard.

**Figure 2:**
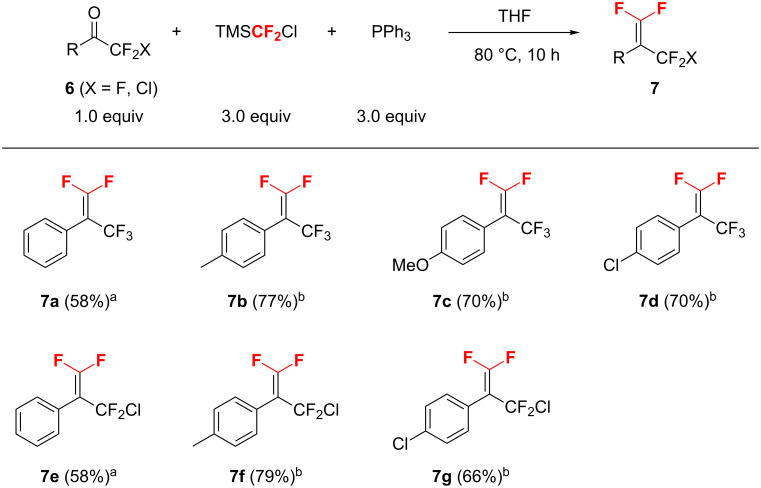
*gem*-Difluoroolefination of activated ketones. Reactions were performed on 0.5 mmol scale in a pressure tube. ^a^Yield was determined by ^19^F NMR spectroscopy using PhCF_3_ as an internal standard. ^b^Isolated yield.

Using the conditions shown in Table 1, entry 2 as standard, the olefination of aldehydes with TMSCF_2_Cl was investigated. As shown in Figure 1, a variety of structurally diverse aromatic aldehydes were successfully converted into *gem*-difluoroalkenes **2a**–**g** in moderate to good yields. It should be mentioned that the aromatic aldehydes with substituents such as *t*-butylthio, methoxy, and bromo groups on the phenyl ring showed similar reactivity. Moreover, this approach is also amenable to enolizable aldehydes, for example, *gem*-difluoroolefin **2h** could be obtained in 47% yield. Although a non-activated ketone such as acetophenone is unreactive under similar conditions, activated ketones could undergo this Wittig olefination reaction. Representative results for the olefination at a slightly elevated temperature (80 °C) are shown in Figure 2. A range of aryl trifluoromethyl (**6a**–**d**) and chlorodifluoromethyl aromatic ketones (**6e**–**g**) were readily difluoromethylenated to give the corresponding olefins (**7a**–**g**) in moderate to good yields. It should be mentioned that in all cases, the formation of *gem*-difluoroolefins was accompanied by the formation of Ph_3_PF_2_ (δ −41.2, d, ^1^*J*_P-F_ = 668 Hz) [25], HCF_2_Cl, fluorotrimethylsilane, and some unidentified byproducts in variable yields (for details, see Supporting Information File 1).

**Scheme 5:**
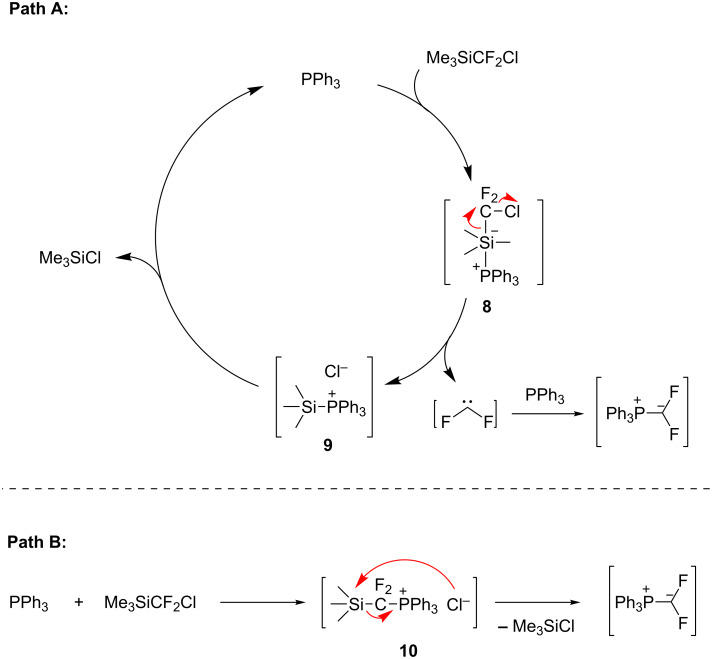
Plausible mechanisms for the formation of difluoromethylene triphenylphosphonium ylide from TMSCF_2_Cl and PPh_3_.

As previously reported, the key mechanistic issue of this Wittig-type reaction is the formation of the presumed difluoromethylene triphenylphosphonium ylide [19–25]. Initially it was speculated that it were trace amounts of nucleophilic impurities (such as chloride ions) that initiated the fragmentation of TMSCF_2_Cl to release :CF_2_ [31], which combined with PPh_3_ to form the ylide. However, the experiment at room temperature showed that PPh_3_ could significantly accelerate the decomposition of TMSCF_2_Cl, which indicated that PPh_3_ should have participated in the activation of TMSCF_2_Cl. Consequently, two plausible mechanisms are proposed (Scheme 5): one is the initial activation of the C–Si bond by PPh_3_ (Path A), the other is the initial activation of the C–Cl bond by PPh_3_ (Path B). In Path A, PPh_3_ firstly coordinates the silicon atom of TMSCF_2_Cl to form activated penta-coordinated silicon species **8** [41] and activates both the C–Si and the C–Cl bond. Next, the release of CF_2_ leads to silylphosphonium salt **9**. Finally, the fragmentation of **9** occurs to give TMSCl with regeneration of PPh_3_; meanwhile, the trapping of :CF_2_ by PPh_3_ gives the ylide. In Path B, a phosphonium salt **10**, which is formed via a single-electron transfer (SET) mechanism, undergoes a chloride ion-promoted desilylation reaction to afford Ph_3_P=CF_2_ [42–43]. However, we could not rule out the possibility of chloride ion-activation in these processes due to the involvement of intermediates **9** and **10** in the proposed mechanisms.

## Conclusion

In conclusion, a robust difluoromethylenation reagent (chlorodifluoromethyl)trimethylsilane (TMSCF_2_Cl) has been prepared via a relatively environmentally benign method and has been successfully used in the Wittig difluoroolefination. Similar as many other Wittig-type *gem*-difluoroolefination reactions in the presence of PPh_3_, the reaction of TMSCF_2_Cl with aldehydes and activated ketones is effective. Comparative reactions with TMSCF_2_Br and TMSCF_3_ under similar conditions failed to give the *gem*-difluorinated olefins, which indicate that the halo-substituent of TMSCF_2_X can influence the reactivity of these fluorinated silanes in difluoromethylene transfer reactions. Further research on the synthetic application of TMSCF_2_X (X = F, Cl, and Br) is currently underway.

## Supporting Information

Full experimental details (difluoromethylation of *O*, *S*, and *N*-nucleophiles and *gem*-difluoroolefination of carbonyl compounds with TMSCF_2_Cl) and compound characterization data are given.

File 1 Experimental procedures and characterization data.
