# Supplementary material for: Deoxygenative gem-difluoroolefination of carbonyl compounds with (chlorodifluoromethyl)trimethylsilane and triphenylphosphine
Source: Beilstein J Org Chem. 2014 Feb 6;10:344–51. doi: 10.3762/bjoc.10.32 (PMC3943835; doi:10.3762/bjoc.10.32)
Supplement: File 1 — Experimental procedures and characterization data. [file Beilstein_J_Org_Chem-10-344-s001.pdf]

Supporting information

for

Deoxygenative *gem*-difluoroolefination of carbonyl compounds with (chlorodifluoromethyl)trimethylsilane and triphenylphosphine

Fei Wang, Lingchun Li, Chuanfa Ni and Jinbo Hu*

Address: Key Laboratory of Organofluorine Chemistry, Shanghai Institute of Organic Chemistry, Chinese Academy of Sciences, 345 Ling-Ling Road, Shanghai 200032, China

Email: Jinbo Hu* - jinbohu@sioc.ac.cn

*Corresponding author

Experimental procedures and characterization data

Table of Contents

1. General information……………………………………………………………..………S2

2. Difluoromethylation of *O*, *S*, and *N*-nucleophiles with $TMSCF_2Cl$……………..… ..S2

3. *gem*-Difluoroolefination of carbonyl compounds with $TMSCF_2Cl$…………...…...…S4

4. ^{19}F NMR spectra of the crude reaction mixture (PhCHO) ……...……………....…S8

5. References……………………....………………....………………....……….……S9

1. General information:

Unless otherwise mentioned, solvents and reagents were purchased from commercial sources and used as received. ^{1}H, ^{13}C and ^{19}F NMR spectra were recorded on 500 MHz, 400 MHz or 300 MHz NMR spectrometer. ^{1}H NMR chemical shifts were determined relative to internal $(CH_3)_4Si$ (TMS) at δ 0.0 or to the signal of a residual protonated solvent: $CDCl_3$ δ 7.26. ^{13}C NMR chemical shifts were determined relative to internal TMS at δ 0.0. ^{19}F NMR chemical shifts were determined relative to $CFCl_3$ at δ 0.0. Mass spectra were obtained on a mass spectrometer. High-resolution mass data were recorded on a high-resolution mass spectrometer in the EI, ESI or MALDI mode.

2. Difluoromethylation of *O*-, *S*-, and *N*-nucleophiles with $TMSCF_2Cl$

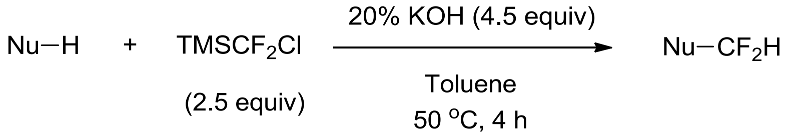

General procedures:

Under a N_2 atmosphere, compounds **S1**, **S3**, or **S5** (1.0 mmol), KOH (0.8 mL, 25 wt %, ca. 4.5 mmol) were added into a pressure tube at room temperature (rt). The reactant mixture was cooled to −78 °C, then $TMSCF_2Cl$ (395 mg, 2.5 mmol) in toluene (2.0 mL) was added. The tube was sealed, and heated at 50 °C for 4 h (**NOTE**: The reaction conditions were not optimized). After being cooled to rt, the reaction mixture was quenched by adding water (5 mL), and extracted with Et_2O (3 × 15 mL). The organic layers were dried over anhydrous $MgSO_4$, concentrated in vacuo, and purified by column chromatography (silica gel; petroleum ether/ethyl acetate) to afford the desired products **S2**, **S4**, **S6,** or **S7** (see Table S-1). All the characterization data were in consistence with the previous report [1].

Table S-1: Difluoromethylation of *O*-, *S*-, and *N*-nucleophiles using $TMSCF_2Cl$[a].

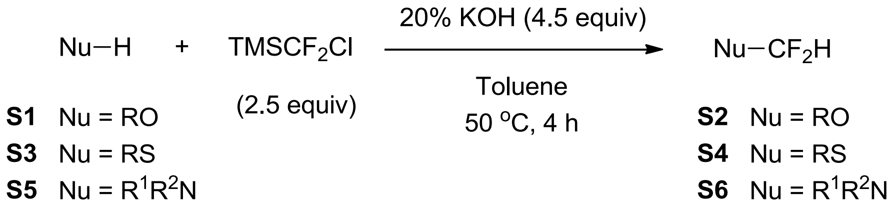

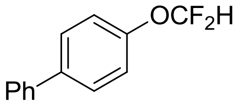

S2a 86%

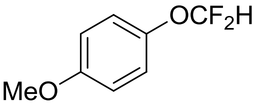

S2b (83%)[b]

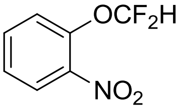

S2c 97%

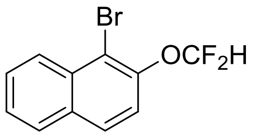

S2d 83%

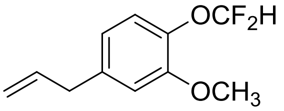

S2e 82%

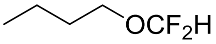

S2f (85%)[b]

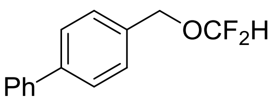

S2g 52%

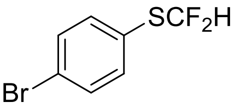

S4a 88%

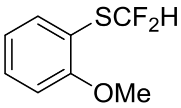

S4b 96%

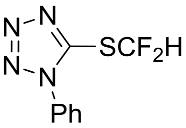

S4c 99%

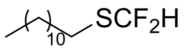

S4d 97%

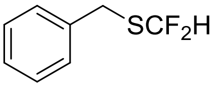

S4e 72%

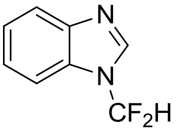

S6a 50%

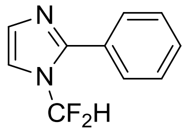

S6b 63%

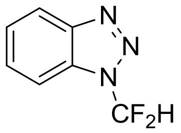

S6c 68%

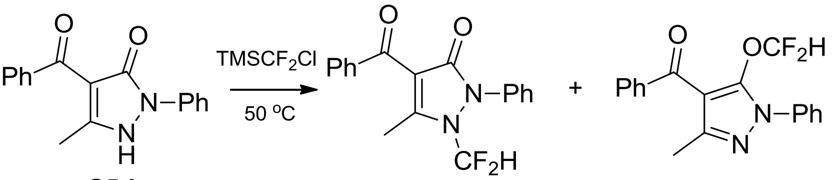

S5d **S6d** 0% **S7d** 49%

[a]All reactions were performed on 1.0 mmol scale in a sealed tube. Yields given refer to the isolated yields. [b]Yields in parentheses were determined by ^{19}F NMR spectroscopy using $PhCF_3$ as an internal standard.

3. *gem*-Difluoroolefination of carbonyl compounds with $TMSCF_2Cl$

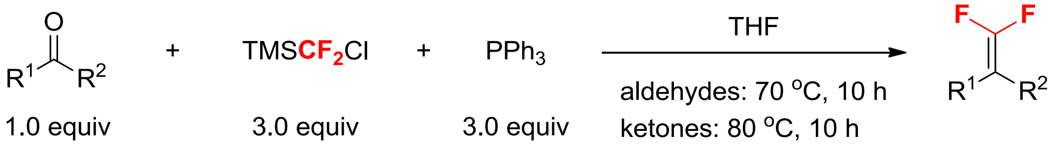

Typical procedures:

To a solution of 1-naphthaldehyde (**1a**) (78 mg, 0.5 mmol) in THF (2 mL), Me_3SiCF_2Cl (237 mg, 1.5 mmol) and triphenylphosphine (393 mg, 1.5 mmol) were added at room temperature. The reaction tube was sealed, and the mixture was heated to 70 °C and stirred for 10 h. The reaction was quenched with water (5 mL), then extracted with Et_2O (20 mL) twice, the combined organic phase was dried over $MgSO_4$. After the removal of solvents under vacuum, the crude product was further purified by silica gel column chromatography to give product **2a** as a colorless liquid. Yield: 59% (56 mg).

1-(2,2-Difluorovinyl)naphthalene (**2a**) [2]

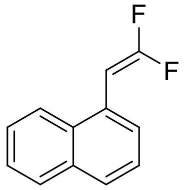

Colorless liquid. ^{1}H NMR: δ 8.03−7.37 (m, 7H), 5.68 (dd, *J* = 22.8 Hz, *J* = 2.5 Hz, 1H). ^{19}F NMR: δ −83.2 (dd, *J* = 29.6 Hz, *J* = 3.6 Hz, 1F), −84.9 (dd, *J* = 29.7 Hz, *J* = 24.5 Hz, 1F). MS (EI, m/z, %): 190 (M^+, 100.00), 170 (71.43), 189 (47.17).

2-(2,2-Difluorovinyl)naphthalene (**2c**) [3]

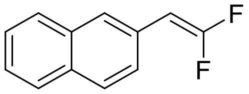

Colorless liquid. ^{1}H NMR: δ 7.88−7.72 (m, 4H), 7.55−7.43 (m, 3H), 5.43 (dd, *J* = 27.0 Hz, *J* = 3.9 Hz, 1H). ^{19}F NMR: δ −81.8 (dd, *J* = 31.1 Hz, *J* = 26.1 Hz, 1F), −83.6 (dd, *J* = 30.6 Hz, *J* = 4.2 Hz, 1F). MS (EI, m/z, %): 190 (M^+, 100.00), 115 (48.05),

143 (30.63).

4-(2,2-Difluorovinyl)biphenyl (**2d**) [4]

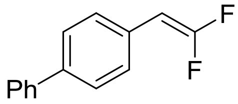

Colorless liquid. ^{1}H NMR: δ 7.63−7.51 (m, 4H), 7.49−7.32 (m, 5H), 5.33 (dd, *J* = 25.8 Hz, *J* = 3.6 Hz, 1H). ^{19}F NMR: δ −82.4 (dd, *J* = 30.7 Hz, *J* = 25.6 Hz, 1F), −84.3 (dd, *J* = 28.8 Hz, *J* = 3.9 Hz, 1F). MS (EI, m/z, %): 190 (M^+, 100.00), 115 (48.05).

tert-Butyl(2-(2,2-difluorovinyl)phenyl)sulfane (**2e**)

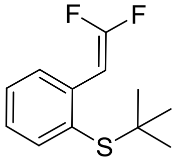

Colorless liquid. IR (film): 3095, 3061, 2964, 2942, 2900, 2864, 1726, 1471, 1458, 1365, 1342, 1236, 1170, 1122, 940, 757, 574 cm^{-1}. ^{1}H NMR: δ 8.25−7.49 (m, 4H), 6.85−6.45 (m, 1H), 2.12−1.44 (m, 9H). ^{19}F NMR: δ −82.2−−82.5 (m, 1F), −83.2−−83.6 (m, 1F). ^{13}C NMR: δ 156.1 (dd, *J* = 297.5 Hz, *J* = 285.2 Hz), 139.2, 136.0 (t, *J* = 7.1 Hz), 128.3, 128.0, 127.9, 126.7, 81.3 (dd, *J* = 32.2 Hz, *J* = 9.9 Hz), 47.6, 30.8. MS (EI, m/z, %): 228 (M^+, 1.75), 57 (100.00), 172 (58.58). HRMS (EI): calcd. for $C_{12}H_{14}SF_2$: 228.0784; Found: 228.0777.

4-(2,2-Difluorovinyl)-1,2-dimethoxybenzene (**2f**) [5]

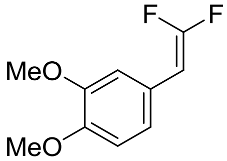

Colorless liquid. ^{1}H NMR: δ 6.89−6.80 (m, 3H), 5.22 (dd, *J* = 26.4 Hz, *J* = 4.2 Hz, 1H), 3.88 (s, 3H), 3.87 (s, 3H). ^{19}F NMR: δ −84.2 (dd, *J* = 36.5 Hz, *J* = 25.9 Hz, 1F), −86.0 (dd, *J* = 36.8 Hz, *J* = 4.4 Hz, 1F). HRMS (EI): calcd. for $C_{12}H_{14}O_2F_2$: 228.0962; Found: 228.0960.

1-Bromo-4-(2,2-difluorovinyl)benzene (**2g**) [6]

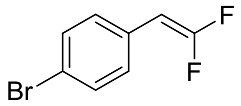

Colorless liquid. ^{1}H NMR: δ 7.46 (d, *J* = 9.0 Hz, 1H), 7.20 (d, *J* = 9.0 Hz, 1H), 5.23 (dd, *J* = 25.8 Hz, *J* = 4.2 Hz, 1H). ^{19}F NMR: δ −81.5 (t, *J* = 28.2 Hz, 1F), −83.4 (dd, *J* = 28.2 Hz, *J* = 3.7 Hz, 1F). MS (EI, m/z, %): 218 (M^+, 100.00), 220 (94.27), 119 (69.41).

(4,4-Difluorobut-3-enyl)benzene (**2h**) [7]

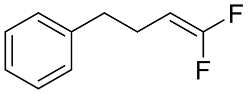

Colorless liquid. ^{1}H NMR: δ 7.36−7.18 (m, 5H), 4.23−4.05 (m, 2H), 2.68 (t, *J* = 7.8 Hz, 2H), 2.35−2.22 (m, 1H). ^{19}F NMR: δ −89.2 (d, *J* = 47.3 Hz, 1F), −91.2 (dd, *J* = 46.8 Hz, *J* = 24.8 Hz, 1F). MS (EI, m/z, %): 168 (M^+, 14.98), 91 (100.00), 73 (13.56).

1-Methyl-4-(perfluoroprop-1-en-2-yl)benzene (**7b**) [8]

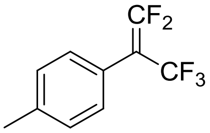

Colorless liquid. ^{1}H NMR: δ 7.22 (s, 4H), 2.38 (s, 3H). ^{19}F NMR: δ −59.6 (dd, *J* = 23.1 Hz, *J* = 10.2 Hz, 3F), −76.0−−76.3 (m, 1F), −77.9−−78.1 (m, 1F). MS (EI, m/z, %): 222 (M^+, 100.00), 133 (47.55).

1-Methoxy-4-(perfluoroprop-1-en-2-yl)benzene (**7c**) [8]

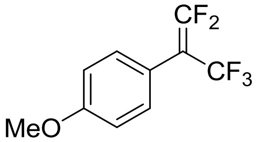

Colorless liquid. ^{1}H NMR: δ 7.24 (d, *J* = 7.2 Hz, 2H), 6.94 (d, *J* = 8.7 Hz, 2H), 3.83 (s, 3H). ^{19}F NMR: δ −59.8 (dd, *J* = 23.4 Hz, *J* = 10.2 Hz, 3F), −76.1−−76.4 (m, 1F), −78.3−−78.3 (m, 1F). MS (EI, m/z, %): 238 (M^+, 100.00), 145 (37.37), 195 (27.80).

1-Chloro-4-(perfluoroprop-1-en-2-yl)benzene (**7d**) [9]

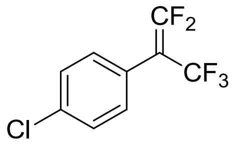

Colorless liquid. ^{1}H NMR: δ 7.41 (d, *J* = 8.4 Hz, 2H), 7.27 (d, *J* = 7.5 Hz, 2H). ^{19}F NMR: δ −59.5 (dd, *J* = 24.0 Hz, *J* = 10.7 Hz, 3F), −74.7–−75.0 (m, 1F), −76.8–−76.9 (m, 1F). MS (EI, m/z, %): 242 (M^+, 100.00), 138 (42.40).

1-(3-Chloro-1,1,3,3-tetrafluoroprop-1-en-2-yl)-4-methylbenzene (**7f**)

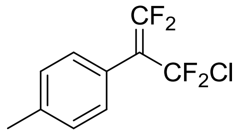

Colorless liquid. IR (film): 3036, 2929, 2873, 1909, 1784, 1732, 1615, 1516, 1333, 1313, 1269, 1228, 1211, 1133, 1033, 966, 871, 814, 790, 613, 512 cm^{-1}. ^{1}H NMR: δ 7.24 (s, 4H), 2.39 (s, 3H). ^{19}F NMR: δ −46.7 (dd, *J* = 32.1 Hz, *J* = 9.0 Hz, 2F), −75.3–−75.6 (m, 1F), −79.1 (dt, *J* = 11.3 Hz, *J* = 10.2 Hz, 1F). ^{13}C NMR: δ 154.6 (ddt, *J* = 305.8 Hz, *J* = 290.2 Hz), 139.5, 130.1 (dd, *J* = 3.1 Hz, *J* = 1.9 Hz), 129.4, 129.3, 123.5 (t, *J* = 1.4 Hz), 95.1 (dtd, *J* = 28.6 Hz, *J* = 28.5 Hz, *J* = 11.2 Hz), 21.1. MS (EI, m/z, %): 238 (M^+, 51.86), 203 (100.00), 133 (55.23). HRMS (EI): calcd. for $C_{10}H_7F_4Cl$: 238.0172; Found: 238.0179.

1-Chloro-4-(3-chloro-1,1,3,3-tetrafluoroprop-1-en-2-yl)benzene (**7g**)

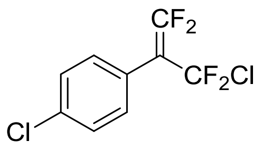

Colorless liquid. IR (film): 1905, 1783, 1733, 1654, 1598, 1495, 1334, 1270, 1224, 1161, 1135, 1095, 1034, 966, 871, 825, 611, 512, 494 cm^{-1}. ^{1}H NMR: δ 7.42 (d, *J* = 8.4 Hz, 2H), 7.30 (d, *J* = 8.1 Hz, 2H). ^{19}F NMR: δ −46.7 (dd, *J* = 31.3 Hz, *J* = 9.6 Hz, 2F), −74.2 (td, *J* = 31.6 Hz, *J* = 9.6 Hz, 1F), −78.0 (dt, *J* = 9.0 Hz, *J* = 9.3 Hz, 1F). ^{13}C NMR: δ 154.5 (dd, *J* = 306.3 Hz, *J* = 291.3 Hz), 135.7, 131.6 (dd, *J* = 3.0 Hz, *J* = 1.5

Hz), 129.3, 128.97, 128.92, 124.8 (dd, J = 2.7 Hz, J = 1.6 Hz). MS (EI, m/z, %): 258 (M^+, 43.59), 223 (100.00), 225 (32.41). HRMS (EI): calcd. for $C_9H_4F_4Cl_2$: 257.9626; Found: 257.9622.

4. ^{19}F NMR spectra of the crude reaction mixture (PhCHO)

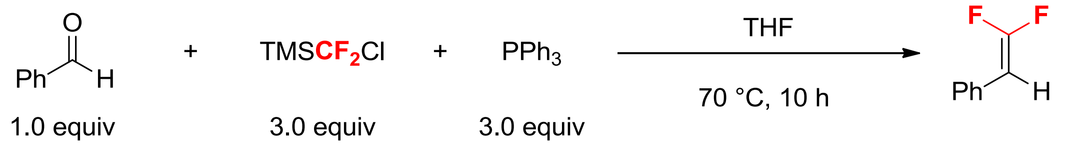

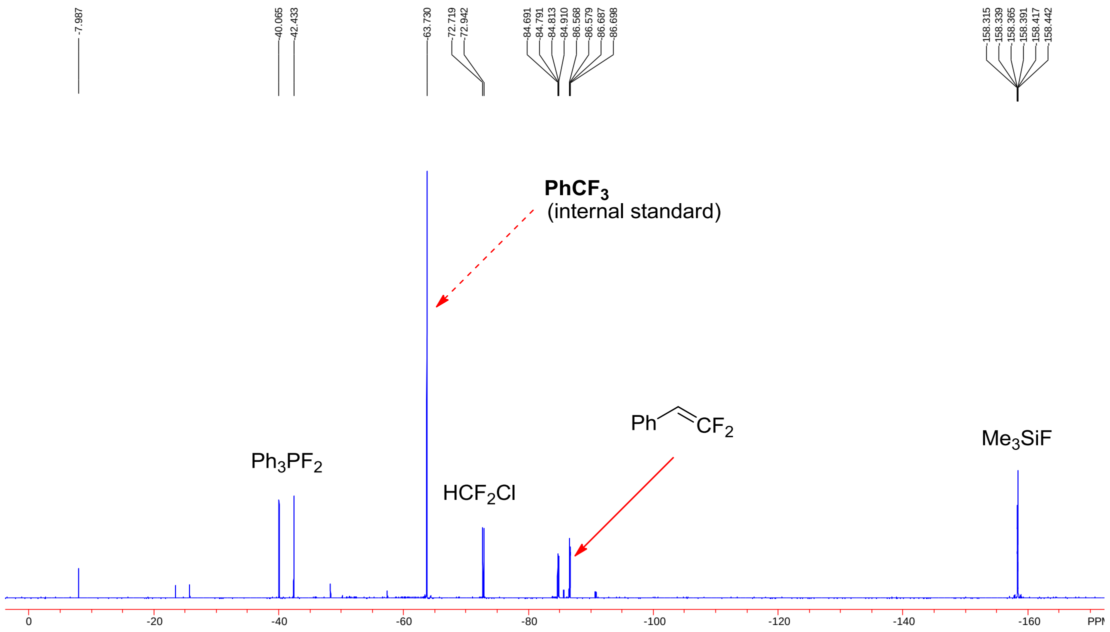

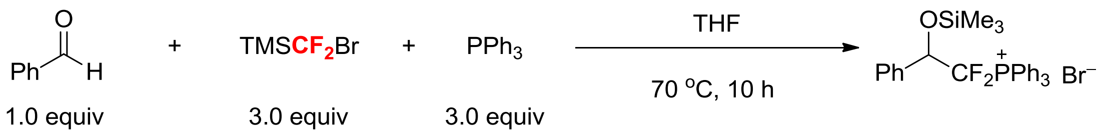

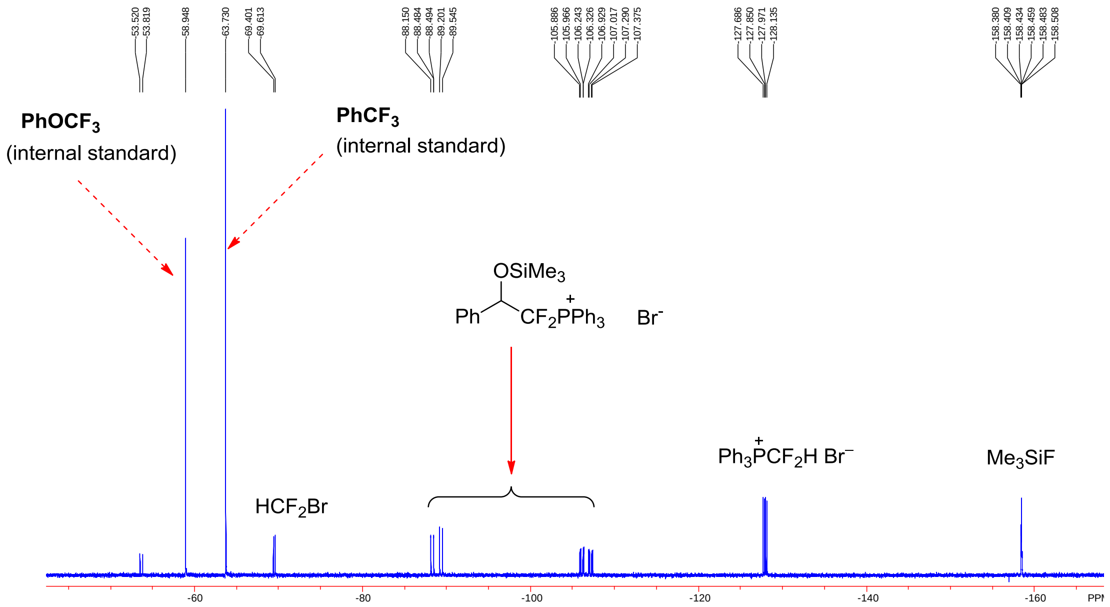

5. References

1. Li, L.; Wang, F.; Ni, C.; Hu, J. *Angew. Chem., Int. Ed.* **2013**, *52*, 12390 – 12394.

2. Nowak, I.; Robins, M. J. *Org. Lett*. **2005**, *7*, 721

3. Edwards, M. L.; Stemerick, D. M.; Jarvi, E. T. *Tetrahedron Lett*. **1990**, *31*, 5571.

4. Zhu, L.; Li, Y.; Zhao, Y.; Hu, J. *Tetrahedron Lett*. **2010**, *51*, 6150.

5. Pohmakotr, M.; Boonkitpattarakul, K.; Ieawsuwan, W.; Jarussophon, S.; Duangdee, N.; Tuchinda, P.; Reutrakul, V. *Tetrahedron* **2006**, *62*, 5973.

6. Nenajdenko, V. G.; Varseev, G. N.; Korotchenko, V. N.; Shastin, A. V.; Balenkova, E. S. *J. Fluorine Chem*. **2003**, *124*, 115.

7. Ichikawa, J.; Jyono, H.; Kudo, T.; Fujiwara, M.; Yokota, M. *Synthesis* **2005**, 39.

8. Morken, P. A.; Burton, D. J. *J. Org. Chem*. **1993**, *58*, 1167.

9. Herkes, F. E.; Burton, D. J. *J. Org. Chem*. **1967**, *32*, 1311.
